# Effect of low doses of actinomycin D on neuroblastoma cell lines

**DOI:** 10.1186/s12943-015-0489-8

**Published:** 2016-01-04

**Authors:** Constanza L. Cortes, Sonia R. Veiga, Eugènia Almacellas, Javier Hernández-Losa, Joan C. Ferreres, Sara C. Kozma, Santiago Ambrosio, George Thomas, Albert Tauler

**Affiliations:** Departament de Bioquímica i Biologia Molecular, Facultat de Farmàcia, Universitat de Barcelona, Avinguda Diagonal 643, 08028 Barcelona, Catalonia Spain; Laboratory of Cancer Metabolism, IDIBELL, Hospital Duran i Reynals, 08908 L’Hospitalet de Llobregat, Barcelona, Catalonia Spain; Pathology Department, Hospital Universitari Vall d’Hebron, Universitat Autónoma de Barcelona, 08035 Barcelona, Catalonia Spain; Laboratory of Cancer Metabolism, Institut Català d’Oncologia, Hospital Duran i Reynals, 08908 L’Hospitalet de Llobregat, Barcelona, Catalonia Spain; Division of Hematology and Oncology, Department of Internal Medicine, College of Medicine, University of Cincinnati, Cincinnati, Ohio 45267 USA; Unit de Biochemistry, Department of Physiological Sciences II, Faculty of Medicine, Campus Universitari de Bellvitge - IDIBELL, University of Barcelona, 08908 L’Hospitalet de Llobregat, Barcelona, Catalonia Spain

**Keywords:** Actinomycin D, Neuroblastoma, Apoptosis, Therapy, SAHA

## Abstract

**Background:**

Neuroblastoma is a malignant embryonal tumor occurring in young children, consisting of undifferentiated neuroectodermal cells derived from the neural crest. Current therapies for high-risk neuroblastoma are insufficient, resulting in high mortality rates and high incidence of relapse. With the intent to find new therapies for neuroblastomas, we investigated the efficacy of low-doses of actinomycin D, which at low concentrations preferentially inhibit RNA polymerase I-dependent rRNA trasncription and therefore, ribosome biogenesis.

**Methods:**

Neuroblastoma cell lines with different p53 genetic background were employed to determine the response on cell viability and apoptosis of low-dose of actinomycin D. Subcutaneously-implanted SK-N-JD derived neuroblastoma tumors were used to assess the effect of low-doses of actinomycin D on tumor formation.

**Results:**

Low-dose actinomycin D treatment causes a reduction of cell viability in neuroblastoma cell lines and that this effect is stronger in cells that are wild-type for p53. *MYCN* overexpression contributes to enhance this effect, confirming the importance of this oncogene in ribosome biogenesis. In the wild-type SK-N-JD cell line, apoptosis was the major mechanism responsible for the reduction in viability and we demonstrate that treatment with the MDM2 inhibitor Nutlin-3, had a similar effect to that of actinomycin D. Apoptosis was also detected in p53^−/−^deficient LA1-55n cells treated with actinomycin D, however, only a small recovery of cell viability was found when apoptosis was inhibited by a pan-caspase inhibitor, suggesting that the treatment could activate an apoptosis-independent cell death pathway in these cells. We also determined whether actinomycin D could increase the efficacy of the histone deacetylase inhibitor, SAHA, which is in being used in neuroblastoma clinical trials. We show that actinomycin D synergizes with SAHA in neuroblastoma cell lines. Moreover, on subcutaneously-implanted neuroblastoma tumors derived from SK-N-JD cells, actinomycin D led to tumor regression, an effect enhanced in combination with SAHA.

**Conclusions:**

The results presented in this work demonstrate that actinomycin D, at low concentrations, inhibits proliferation and induces cell death in vitro, as well as tumor regression in vivo. From this study, we propose that use of ribosome biogenesis inhibitors should be clinically considered as a potential therapy to treat neuroblastomas.

**Electronic supplementary material:**

The online version of this article (doi:10.1186/s12943-015-0489-8) contains supplementary material, which is available to authorized users.

## Background

Neuroblastoma is a malignant embryonal tumor occurring in young children, consisting of undifferentiated neuroectodermal cells derived from the neural crest [1]. It is an aggressive cancer accounting for more than 15 % of all pediatric cancer-related deaths [2]. A main hallmark of neuroblastoma is the variability in clinical outcome, partly due to the multiple cell types forming the tumor mass. Neuroblastoma cell types vary in their degree of differentiation, tumorigenicity and drug sensitivity, having the capability to trans-differentiate into other cell type.

The multiplicity of the genomic alterations described for neuroblastoma indicates that the evolution of this neoplasia involves a complex pattern of oncogene activation and tumor suppressor gene inactivation [3]. About 15 % of the neuroblastoma cases show *MYCN* gene amplification, a genomic aberration used as a negative prognosis indicator [4]. Besides *MYCN* amplification, other aberrations also contribute to tumor progression. For example, upregulation of *MYCN* expression by high expression of the transcription factor E2F1, and/or activation of ALK kinase and/or loss of function of tumor suppressor proteins NF1 and p73, act independently of *MYCN* status [5–7]. Since most neuroblastoma cells are wild-type for p53 (p53^wt^), induction of p53 is viewed as a potential therapeutic approach for this tumor type [8, 9]. Accordingly, most patients with high-risk neuroblastomas, initially respond to genotoxic chemotherapy and local radiotherapy (10). However, no satisfactory treatment is currently available as relapsed neuroblastomas show frequent secondary mutations and represent a serious problem in neuroblastoma management [10, 11].

Inhibition of ribosome biogenesis has been proposed recently as a new therapeutic approach in treating specific cancer types, in particular those driven by dysregulated c-Myc activity [12, 13]. To maintain high proliferation rates, cancer cells need to increase their translational capacity and are addicted to high rates of ribosome biogenesis [13–16]. In this scenario, high c-Myc activity in tumors influences tumor formation, not only by transcriptionally upregulating genes essential for cell cycle progression, but also by increasing global protein translation. c-Myc activity participates in ribosome biogenesis by inducing the expression of ribosomal proteins through RNA polymerase II, by transcriptional upregulating 45S rRNA and 5S rRNA through activation of RNA Pol I and III respectively, as well as by modulating factors essential for the rRNA processing, rRNA transport and ribosome assembly [17]. Importantly, like c-Myc, the specific form of *MYC* in neuroblastoma, N-Myc, also enhances rates of ribosome biogenesis [18]. Impairment of this response leads to the activation of a novel MDM2 checkpoint, leading to stabilization of p53, cell cycle arrest and apoptosis. The severity to which the checkpoint is engaged, appears to be governed by the extent to which cell is dependent on ribosome biogenesis. Given the addiction c-Myc induced tumors to high rates of ribosome biogenesis, we hypothesized that inhibition of ribosome biogenesis could be an selective approach for neuroblastoma therapy [19].

Actinomycin D was the first antibiotic shown to have anti-cancer activity, and is now most commonly used as a treatment for a variety of pediatric tumors, such as Wilms’ tumor, Rhabdomyosarcoma and Ewing’s sarcoma [20–22]. Actinomycin D is a DNA intercalator, which shows preference for GC-rich DNA sequences [23]. As the promoter of 45S ribosomal gene is GC-rich, low concentrations of actinomycin D preferentially inhibit RNA Pol I-dependent trasncription, leading to a disruption of ribosome biogenesis [23]. As a consequence, a preribosomal complex made up of ribosomal proteins RPL5 and RPL11 and non-coding 5S rRNA is redirected from 60S ribosome biogenesis to the binding of MDM2, inhibiting its ubiquitin-ligase activity and promoting the accumulation of p53, cell cycle arrest and apoptosis [24]. Interestingly, actinomycin D also induces cell death in patients with deleted or mutated p53, suggesting the existence of a p53-independent cell death mechanisms [25].

Here we studied the response of neuroblastoma cell lines to low doses of actinomycin D in cell culture and xenograft tumor models. We also tested the combinatory effect of actinomycin D with the p53-independent chemotherapeutic agent suberoylanilide hydroxamic acid, SAHA, which is now in clinical trials for neuroblastomas treatment [26]. Our data highlights the therapeutic potential of actinomycin D and suggests that low doses of this drug could be used in combination with other agents to take advantage of its dependence on p53, but avoid its non-specific effects.

## Results

### Actinomycin D decreases cell viability in a dose- and time-dependent manner

**Fig. 1:**
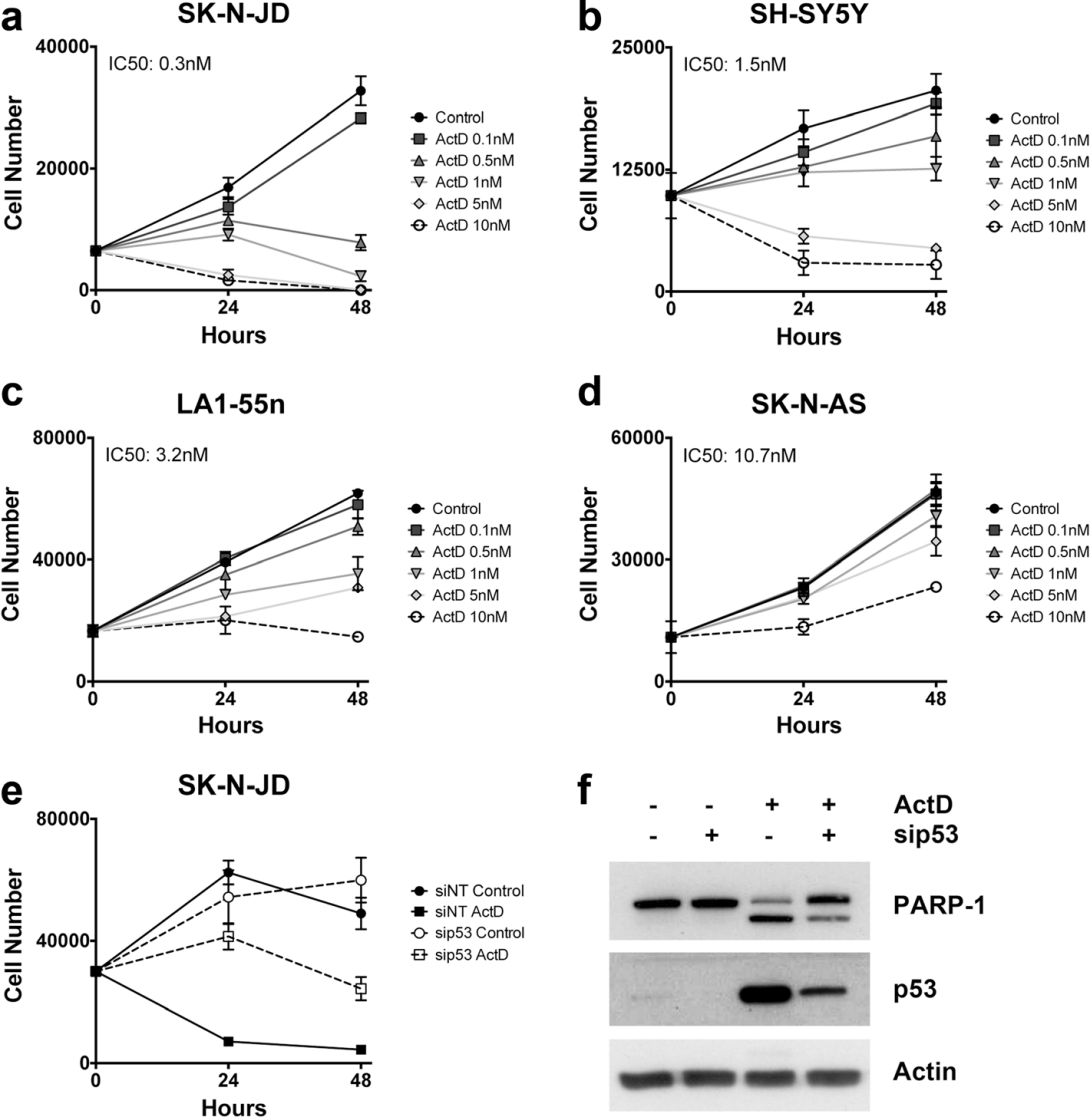
Effect of actinomycin D on cell viability. **a**–**d** Cell lines were treated with the indicated doses of actinomycin D and cell viability was measured after 24 h and 48 h of treatment. IC50 was calculated at 48 h of treatment using the CalcuSyn software (Biosoft Inc.). **e**-**f** SK-N-JD cells were transfected with non-targeting siRNA (siNT) or p53 siRNA for 48 h and treated with the indicated doses of actinomycin D. **e** Cell viability was measured 24 h and 48 h after treatment. **f** Expression of the indicated proteins was determined by Western blot analysis 24 h after treatment with 10 nM of actinomycin D

To assess the impact of Pol I inhibition on neuroblastoma cell viability, we measured the effect of escalating doses of actinomycin D on a representative panel of neuroblastoma cell lines [27]. Concentrations used were in the nM range, which have been shown to inhibit Pol I without affecting Pol II and Pol III activity [23]. Reduction of cell viability occurred in all neuroblastoma cell lines tested after 24 and 48 h of treatment. However, the extent of this effect varied depending on the cell line (Fig. 1). SK-N-JD and SH-SY5Y, two p53-proficient cell lines, presented a complete reduction of cell viability when tested at higher actinomycin D concentrations (Fig. 1a, b). In contrast, LA1-55n and SK-N-AS, two cell lines with either absent or truncated p53, were considerably less responsive to treatment (Fig. 1c, d). These differences would suggest a sensitization to actinomycin D depending on p53 genetic background. To confirm this hypothesis, the expression of p53 was abrogated by siRNA in SK-N-JD cells and we measured the response to actinomycin D. The results confirmed that p53 depleted cells show less sensitivity to actinomycin D than the parental cells (Fig. 1e). Note that p53 depletion reduced the level of PARP-1 cleavage, suggesting that a p53 apoptosis-dependent mechanism is involved on reduction of cell viability after actinomycin D treatment (Fig. 1f).

**Fig. 2:**
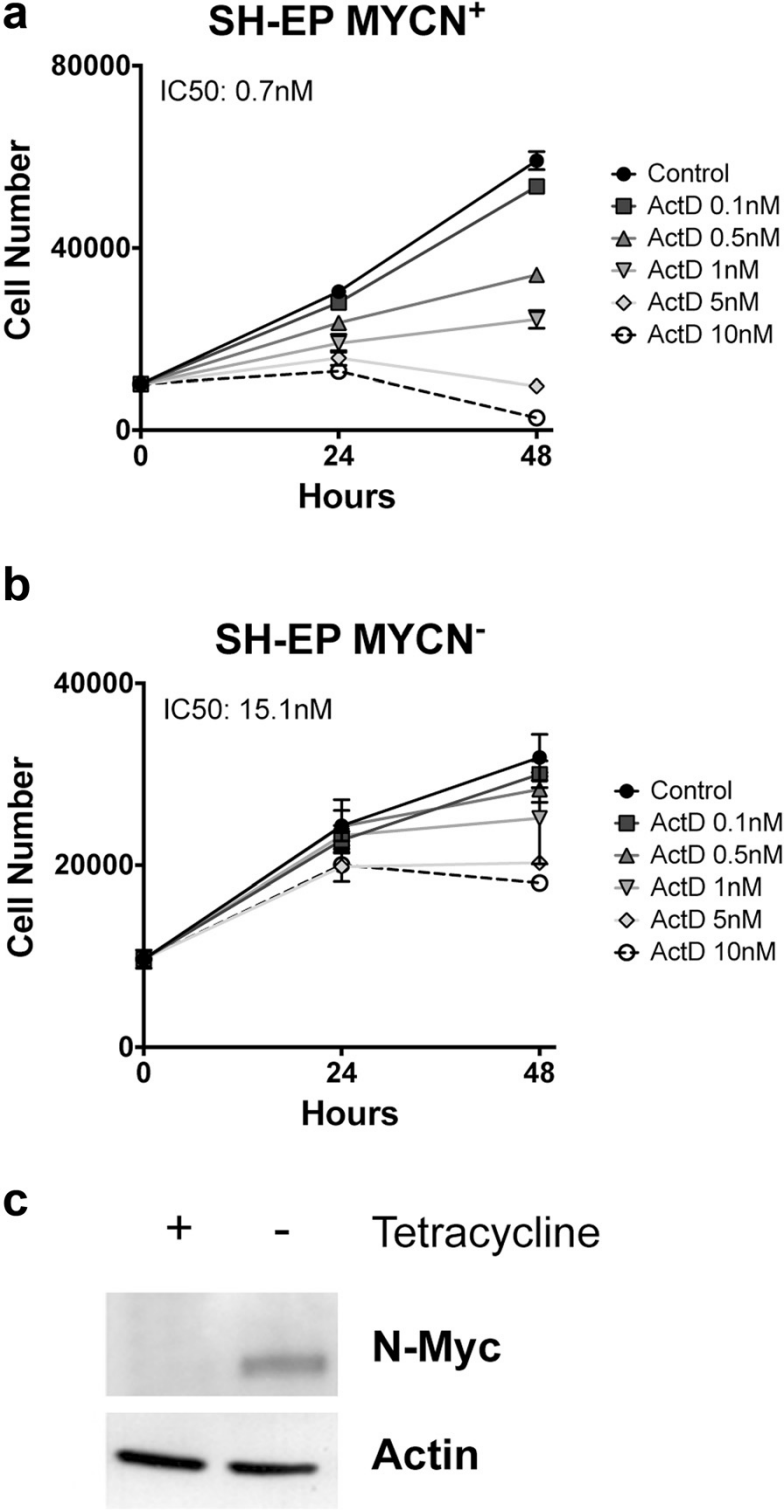
Role of N-Myc on the response to actinomycin D. **a**-**c** SH-EP *Tet*/21n cells were treated in absence (MYCN^+^) or in presence (MYCN^−^) of tetracycline with increasing doses of actinomycin D, and cell viability was measured after 24 and 48 h of treatment. IC50 was calculated at 48 h of treatment using the CalcuSyn software (Biosoft Inc.). **c** N-Myc protein expression was determined by Western blot analysis, 24 h after tetracycline addition

We hypothesized that overexpression of *MYCN* gene, which is often amplified in neuroblastoma, could play a role in actinomycin D response. Consistent with this, in cells with equal p53 status, cells with *MYCN*-amplified genetic background showed more sensitivity to actinomycin D than those that do not present this gene amplification; compare SK-N-JD with SH-SY5Y and LA1-55n with SK-N-AS (Fig. 1). In order to rule out that the differences in response were due to cell type, we further analyzed the effect of N-Myc over-expression in SH-EP *Tet*/21 N cells. This cell line expresses an exogenous *MYCN* gene regulated by a tetracycline repressible promoter [28]. In response to actinomycin D, N-Myc overexpressed SH-EP cells showed a marked reduction of cell viability compared to those in which c-Myc was downregulated by tetracycline induction, suggesting that N-Myc levels sensitize neuroblastoma to actinomycin D treatment (Fig. 2a-c).

Taken together, the results show that low concentrations of actinomycin D cause a reduction of cell viability in neuroblastoma cell lines, this effect is stronger in cells with p53^wt^ genetic background and *MYCN* overexpression appears to enhance actinomycin D sensitivity.

### Actinomycin D induces cell death by apoptosis-dependent and independent mechanisms

**Fig. 3:**
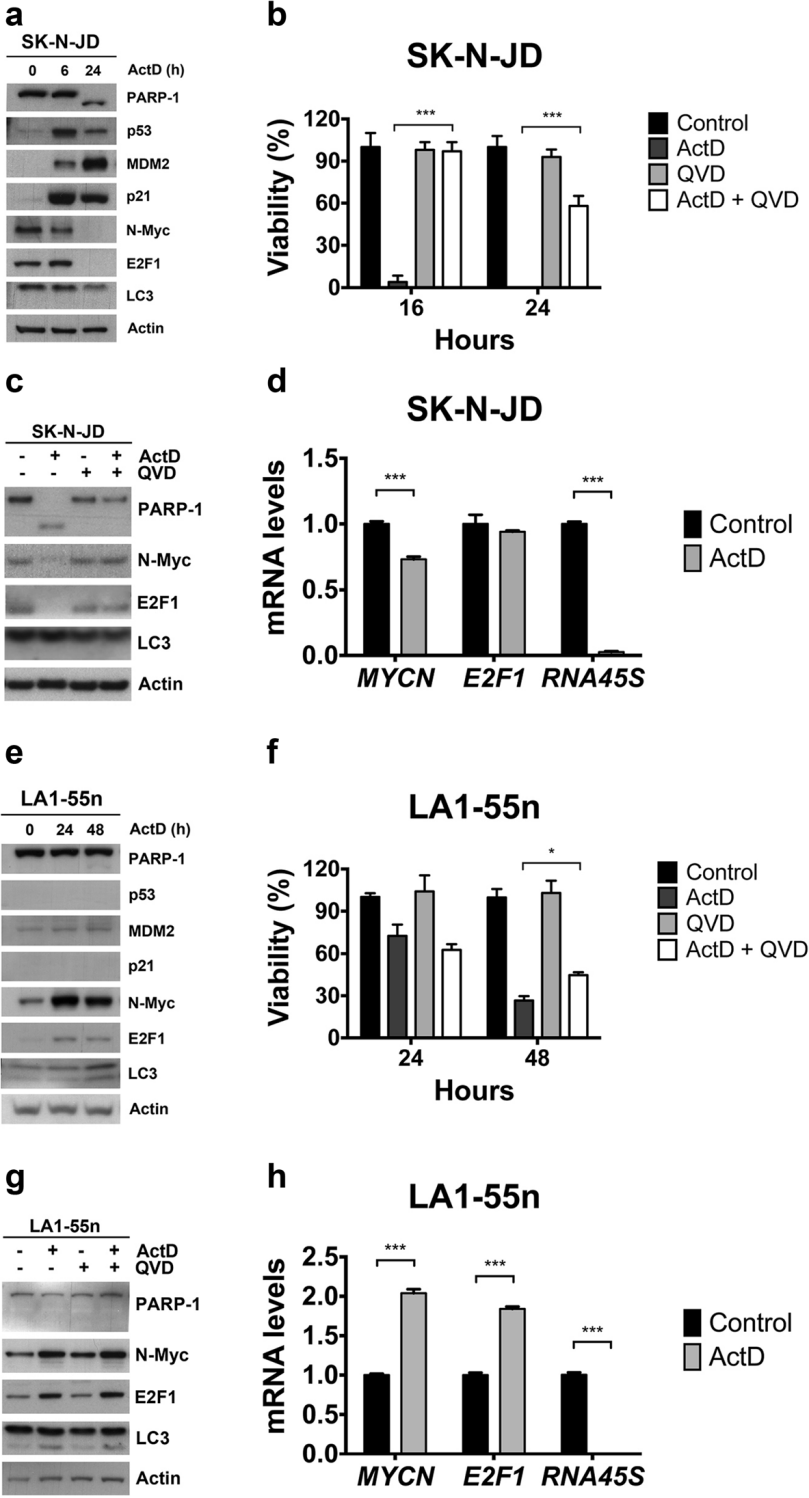
Role of actinomycin D on apoptosis. **a** and **e** Cell lines were treated with 10 nM of actinomycin D and protein expression was determined by Western Blot analysis at the given times. **b** and **f** Cell lines were treated with 10 nM of actinomycin D in presence or absence of 20 μM of Oph-QVD and cell viability was measured at indicated times. **c** and **g** Cell lines were treated with actinomycin D 10 nM in presence or absence of Oph-QVD 20 μM. After 24 h of treatment, the indicated protein expression levels were determined by Western blot analysis. **d** and **h** Cell lines were treated with 10 nM of actinomycin D and levels of the indicated RNAs were measured 24 h after treatment

Previous studies demonstrate that low doses of actinomycin D stabilize p53 by inhibiting MDM2 [24]. Accumulation of p53 due to MDM2 inhibition leads to cell cycle arrest and/or apoptosis [29]. Focusing on the p53^wt^ SK-N-JD and p53-deficient LA1-55n cell lines, we investigated the mechanism by which actinomycin D represses cell viability. Apoptosis, assessed by PARP-1 cleavage, was observed after actinomycin D treatment in the p53^wt^ SK-N-JD cell line (Fig. 3a). The activation of apoptosis correlated with an increase in p53 levels and its transcriptional targets MDM2 and p21, as well as a decrease in N-Myc and E2F1 levels (Fig. 3a). Inhibition of apoptosis by the presence of the pan-caspase inhibitor QVD-Oph, rescued the reduction of cell viability induced by actinomycin D at 16 h and partially at 24 h, implying a key role of caspases in this process (Fig. 3b). Protein analysis showed that inhibition of caspase activity blocked N-Myc and E2F1 degradation, strongly implying that both proteins could be, directly or indirectly, downstream targets of caspases (Fig. 3c). Note that N-Myc RNA decreased after actinomycin D treatment and 45S rRNA levels, as a measure of pol I activity, were almost undetectable (Fig. 3d).

PARP-1 cleavage was not detected following actinomycin D treatment of p53-deficient LA1-55n cells (Fig. 3e). However, a small, but significant, recovery of cell viability was found when QVD-Oph was added to actinomycin D treated cells, suggesting that apoptosis could also be induced in this cell line (Fig. 3f). The involvement of apoptosis was confirmed by flow cytometry analysis. Treatment with actinomycin D induced an increase in cell death, manifested by accumulation of sub-G1 cell debris population and a significant increase in G2 population. The presence of the pan-caspase inhibitor, QVD-Oph totally abrogated the increase of the number of cells in the sub-G1 cell debris population, completely abolishing this response in the SK-N-JD cell line (Additional file 1: Figure S1). Moreover, in contrast to p53^wt^ SK-N-JD cells, E2F1 and N-Myc protein levels rose after actinomycin D treatment of p53-deficient LA1-55n cells, with no further effect of QVD-Oph treatment (Fig. 3g). At this time, N-Myc and E2F1 mRNA levels also increased suggesting that the action of actinomycin D occurs at mRNA level, either through increased transcription or mRNA stability (Fig. 3h).

Autophagy has been described as a mechanism of cell death in several settings including neuroblastoma [27]. Autophagy was analyzed by LC3-I conversion to LC3-II, the LC3-lipidated form bound to autophagosomes [30]. Increased conversion of LC3-I to LC3-II was observed in LA1-55n cells but not in SK-N-JD, suggesting that autophagy could play a role in viability loss in cells with a p53^−/−^ genetic background (Fig. 3c, g). Overall, these results suggest that apoptosis is the major cell death mechanism triggered by actinomycin D treatment in p53^wt^ cell lines and, to a less extent, in p53-deficient cell lines.

### Activation of p53 is responsible for the effects of actinomycin D in p53^wt^ SK-N-JD neuroblastoma cell line

**Fig. 4:**
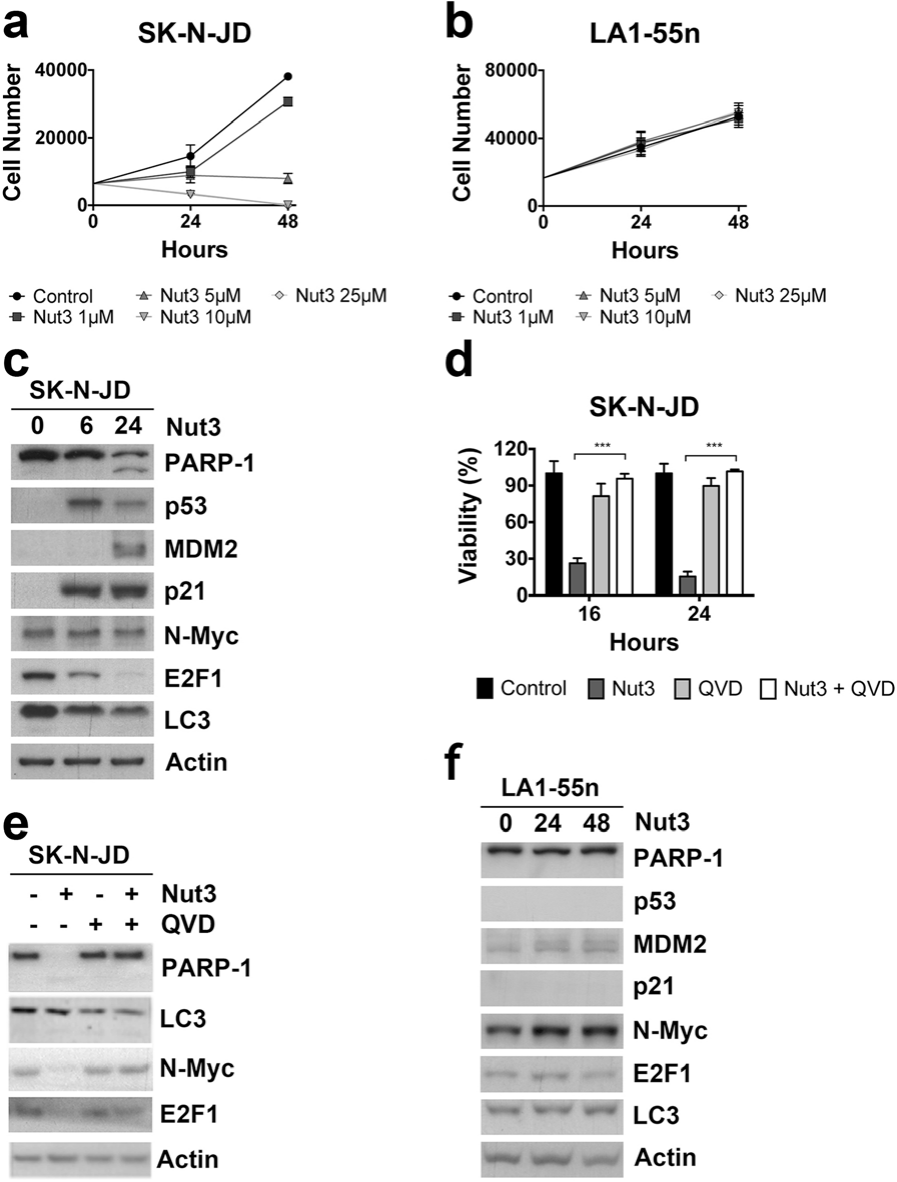
Effect of nutlin-3. **a** and **b** Cell lines were treated with the indicated doses of nutlin-3 and cell viability was measured after 24 h and 48 h of the treatment. **c** and **f** Cell lines were treated with 10 μM of nutlin-3 and expression of the indicated proteins was determined by Western Blot analysis after the given treatment times **d** SK-N-JD cells were treated with 10 μM of nutlin-3, in presence or absence of 20 μM of Oph-QVD. Cell viability was measured at the indicated times. **e** SK-N-JD cells were treated (+) or not (−) with 10 μM of nutlin-3 in the presence (+) or absence (−) of 20 μM of Oph-QVD. Expression of the indicated proteins was determined by Western Blot analysis 24 h after treatment

To further analyze the contribution of p53 on neuroblastoma cell death, we investigated the effect of Nutlin-3 on cell viability. Nutlin-3 specifically activates p53 expression by inhibiting the MDM2-p53 interaction. Escalating doses of Nutlin-3 strongly reduced viability in p53^wt^ SK-N-JD cells at 24 and 48 hrs after treatment and, while no effect was found in the cell line lacking functional p53, LA1-55n (Fig. 4a). Similarly, as observed above for actinomycin D, the activation of apoptosis in SK-N-JD cells, correlated with the stabilization of p53 and its transcriptional targets MDM2 and p21, as well as a decrease in N-Myc and E2F1 levels (Fig. 4b). The inhibition of apoptosis by QVD-Oph treatment completely rescued the effect of Nutlin-3 on cell viability as well as E2F1 and N-Myc protein expression (Fig. 4c, d). Neither apoptosis nor changes of MDM2 and N-Myc expression levels were observed Nutlin-3-resistent cells LA1-55n cells (Fig. 4e). Results support that the activation of p53, per se, is responsible for the effects of actinomycin D found on SK-N-JD proliferation.

### Actinomycin D synergizes with SAHA to decrease cell viability

**Fig. 5:**
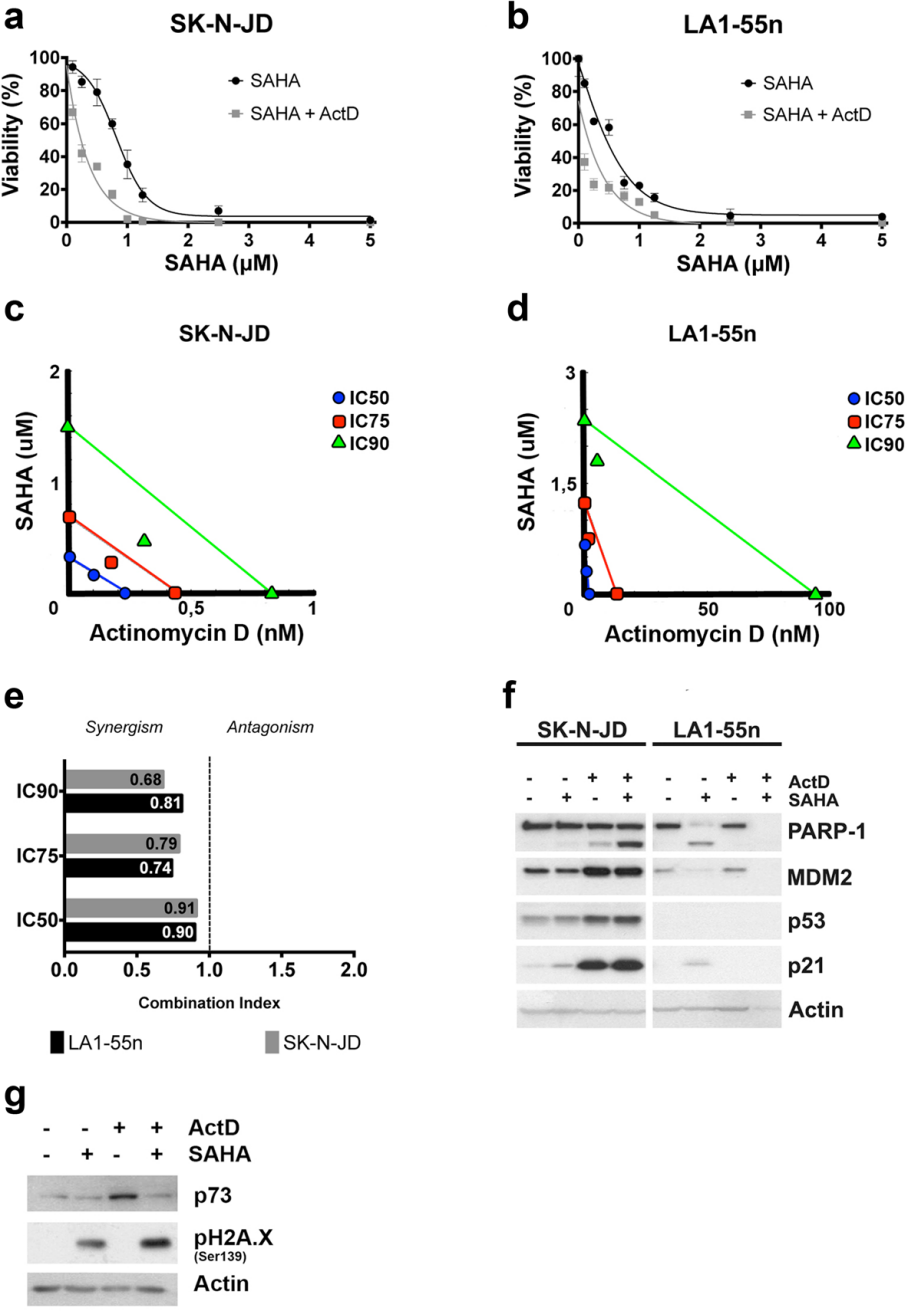
Effect of the combination of Actinomycin D with SAHA in vitro. **a** and **b** Cells were treated for 48 h with the indicated concentrations of SAHA in presence or absence of actinomycin D at 0.25 nM (SK-N-JD) or 2.5 nM (LA1-55n) and cell viability was measured. **c** and **d** Isobolograms representing actinomycin D and SAHA interaction analyzed by the Chou-Talalay median effect method. The additivity line is the one uniting each drug concentration needed to inhibit cell growth by 50 % (IC50), 75 % (IC75) or 90 % (IC90). The colored shapes under this line denote synergism. **e** Graphic representation of IC values at IC50, IC75 and IC90. **f** Cells were treated (+) or not (−) with actinomycin D in the presence (+) or absence (−) of SAHA for 48 h. Concentrations used were 0.3 nM actinomycin D and 0.7 μM SAHA in SK-N-JD cells, and 1 nM actinomycin D and 1 μM SAHA in LA1-55n cells. Protein expression was determined by Western blot analysis. **g** LA1-55n cells were treated (+) or not (−) with 0.1 nM of actinomycin D in the presence (+) or in the absence (−) of 1 μM SAHA. The indicated proteins expression was determined after 24 h after treatment by Western blot analysis

Preclinical and phase I/II clinical trials have provided the basis to appraise histone deacetylase inhibitors as treatment for cancer therapy, including neuroblastoma [26, 31]. Considering this, we examined the effect of SAHA in combination with actinomycin D on neuroblastoma cell viability. Treatment with increasing doses of SAHA, in combination with actinomycin D at low concentrations, led to a more pronounced decrease in cell viability compared with treatment with SAHA alone both, in SK-N-JD and LA1-55n, neuroblastoma cells lines (Fig. 5a, b). Synergy was also tested by the combination index isobologram method (35). Simultaneous exposure to increasing doses of SAHA and actinomycin D showed a CI lower than 1 which implies a synergistic inhibitory effect of the drugs on neuroblastoma cell lines viability (Fig. 5c-e). The effect of the drug combination on apoptosis was also determined by PARP-1 cleavage. Treatment with both drugs augmented PARP-1 cleavage suggesting an increase in cell death by apoptosis (Fig. 5f). Interestingly, the increase of PARP-1 cleavage did not correlate with an increase of the p53 levels, or its target genes p21 and MDM2 suggesting that the synergism occurs independently of p53-induced apoptosis. These results demonstrate that actinomycin D synergizes with SAHA, with the latter apparently activating apoptosis in a p53-independent manner.

The p53 family member, p73, has an important role in apoptosis in settings where p53 is absent [32–34]. To investigate whether p73 could be involved in the response to actinomycin D, alone or in combination with SAHA in 53-deficient cells, levels of p73 were measured in LA1-55n cell lines. Actinomycin D treatment alone increased p73 expression, which was abrogated after SAHA treatment suggesting that p73 expression could be responsible for apoptosis in 53-deficient cells, but not for the SAHA synergism on cell death (Fig. 5g). Since actinomycin D acts preferentially by intercalating into GpC rich double-stranded DNA, we also investigated the activation of DNA double strand break response after such treatments. Phosphorylation of H2A.X at serine 139 was not detected at low doses of actinomycin D discarding this possibility. However in contrast, high levels of γ- H2AX were found in all SAHA treatment conditions, as has been recently reported [35]. This effect was observed after 16 h of treatment (Additional file 2: Figure S2).

### Actinomycin D alone or combined with SAHA causes regression of SK-N-JD derived tumors

**Fig. 6:**
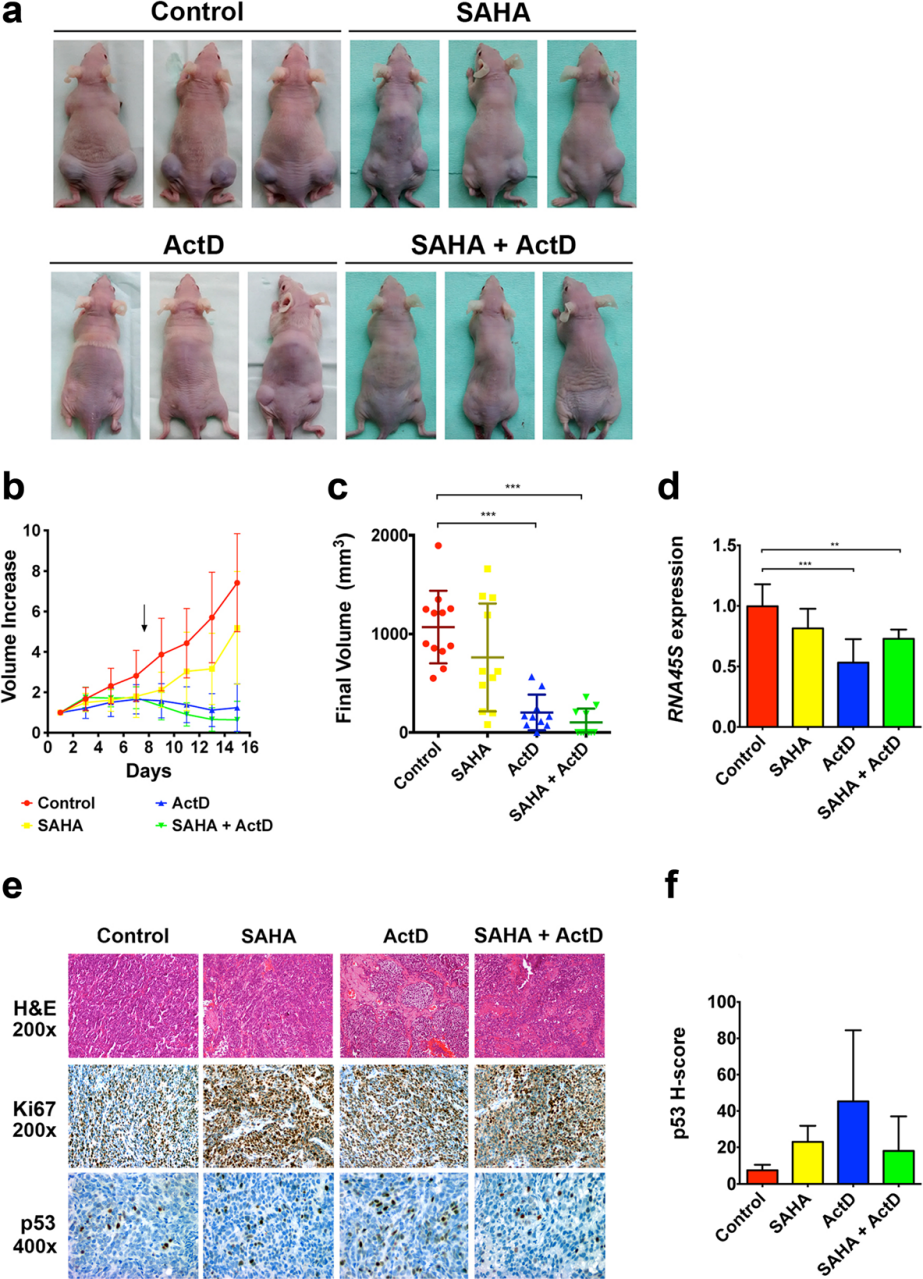
Effect of the combination of Actinomycin D with SAHA in vivo. **a** Tumor size observed in mice after 14 days of each treatment. **b** Tumor volume increase over time, expressed as fold increase over the initial tumor volume. Error bars show ± SEM of at least 10 tumor replicates on eight different mice for each treatment. **c** Final volume of each of the tumors analyzed. **d** RNA levels of *45S* rRNA was measured after 14 days of each treatment. Values represent the average ± SEM of three different tumors for each treatment. **e** Hematoxylin and eosin (H&E), Ki67 and p53 stainings were obtained after 14 days of indicated treatment. **f** Graphic representation of the p53 staining values

The in vitro observations of the ability of actinomycin D to induce cell death and cell cycle arrest was evaluated in vivo by testing its effect on subcutaneously-implanted SK-N-JD derived neuroblastoma tumors. SK-N-JD cells were subcutaneously transplanted into athymic nude mice and tumor pieces of similar size were engrafted in both flanks of recipient mice. Mice bearing the tumor were treated for 15 days with either actinomycin D, SAHA, the combination of actinomycin D and SAHA, or placebo. Treatment with actinomycin D alone or in combination with SAHA, resulted in a delay in tumor growth (Fig. 6a, b). After 14 days of treatment, actinomycin D reduced tumor volume ~ 80 % compared with the vehicle, while SAHA decreased tumor volume by a 30 % (Fig. 6c). The combination of actinomycin D with SAHA reduced tumor volume by 90 % (Fig. 6c). Most significantly, the combination resulted in complete regression of tumors in 7 out of 12 mice. Chronic administration of all drugs was tolerable, with an average weight loss above 12 % at the end of the experiment. 45S rRNA levels were lowered in actinomycin D conditions implying a direct effect of this drug on the tumor (Fig. 6d). Hematoxylin and eosin (H&E) staining of tumor sections revealed an undifferentiated phenotype, depicted by a fibrous stroma (Fig. 6e). No significant changes on proliferative parameters such as Ki67 or mitotic index were observed; however higher p53 levels were observed in some of the sections of actinomycin D treated tumors (Fig. 6e, f). Overall these results show that actinomycin D, alone or in combination with SAHA, leads to tumor regression in our mouse mode, strongly suggesting a potent role for this drug in treatment of human neuroblastoma.

## Discussion

Current therapies for high-risk neuroblastoma are insufficient, resulting in high mortality rates and high incidence of relapse [36]. With the intent to find new therapeutic approaches for this cancer, we investigated the utility of actinomycin D as an inhibitor of ribosome biogenesis in the treatment of neuroblastoma tumors. The results presented in this work demonstrate that actinomycin D, at low concentrations, inhibits proliferation and induces cell death in vitro, as well as tumor regression in vivo. From this study, we propose that use of ribosome biogenesis inhibitors should be clinically considered as a potential therapy to treat neuroblastomas.

Although all the neuroblastoma cell types were sensitive to actinomycin D, the extent of the response was different depending on their p53 genetic status. This agrees with the finding that actinomycin D, at low concentrations, has been described as a potent activator of the p53 pathway. In this regard, we show that actinomycin D treatment has similar features to the MDM2 inhibition by Nutlin-3 in p53-functional SK-N-JD. The results presented here agree with those of others showing that sensitivity to Nutlin-3 was highly predictive in the absence of p53 mutation [37]. It is known that actinomycin D disrupts ribosome biogenesis, redirecting the pre-ribosomal complex RPL11/RPL5/5SrRNA from assembly into nascent 60S ribosomes to the binding and inhibition HDM2, resulting in p53 stabilization [24]. In this context, we found that actinomycin D increases on p53 protein levels and induces the accumulation of the p53 target genes p21 and MDM2. Interestingly, the increase of MDM2 levels at 24 h correlated inversely with the reduction of p53, suggesting the activation of a feed-back loop, as has been previously reported [29]. Apoptosis was the major mechanisms responsible for cell death in the p53^wt^ SK-N-JD neuroblastoma cells and contributes to N-Myc and E2F1 protein degradation.

The fact that actinomycin D was also able to repress cell growth in p53-deficient LA1-55n cells implies the involvement of p53-independent mechanism. Although it was not detected by PARP-1 cleavage analysis, we have shown by flow cytometry analysis that apoptosis was induced after actinomycin D treatment. Apoptotic function of actinomycin D in p53-deficient cells could be a consequence of the activation of p73, a homologue of p53. It is known that p73 binds to the N-terminal hydrophobic pocket of MDM2 and MDM2 inhibits p73 transcriptional activity [33, 38]. Actinomycin D could prevent p73-MDM2 interaction, resulting in p73-dependent apoptosis, in an analogous manner as it has been reported in p53-null neuronal cells after Nutlin-3 treatment [33, 34]. Moreover, the minor effect of pan-caspase inhibitor QVD-Oph on restoring cell viability after actinomycin D treatment indicates that apoptotic-independent mechanisms are mainly responsible for the effect of actinomycin D in these cells. In this regards, a strong G_2_-M cell cycle arrest was detected after actinomycin D treatment, similar to that previously reported [39]. Furthermore, our data indicate that actinomycin D activates autophagy. While autophagy is mainly considered a cell survival mechanism, its activation can induce neuroblastoma cell death under certain conditions [27, 40]. Taken together our results argue for an important p53-independent cell death component induced by actinomycin D in p53 deficient-neuroblastomas, similar to what has been found in chronic lymphocytic leukemia [25].

About 15 % of the neuroblastoma present *MYCN* amplification, an indicator of bad prognosis [4]. *MYCN*, like other members of the MYC family, has been described as a driver of anabolic cellular processes, including ribosome biogenesis. I would be expected that *MYCN* overexpressing tumors would have elevated rates of ribosome biogenesis, and their malignancy dependent on this process as has been described in c-Myc-driven tumors model of B-cell lymphoma [19]. In agreement with this, our results show that higher N-Myc levels sensitize neuroblastoma to actinomycin D. In a wild-type p53 genetic background, the stronger effect of actinomycin D in *MYCN* amplified context should result in the activation of RPL5/RPL11/5S rRNA-MDM2-p53 checkpoint. Our data agrees with previous findings that report a direct correlation between *MYCN* status and the response to the MDM2-p53 antagonist, Nutlin-3 [41]. Similar to the use of DNA damaging agents, which target cancer cells with high replicative rates, drugs that disrupt ribosome biogenesis, such as actinomycin D could be exploited to induce selective apoptosis in tumors characterized by high rates of ribosome biogenesis, such as Myc driven tumors.

Actinomycin D has been used clinically for over 50 years for the treatment of children and adult cancer. As part of a multimodal therapy, actinomycin D is a key component in the treatment of Wilms tumor, Ewing’s sarcoma and rhabdomyosarcoma [42]. Administration of actinomycin D to the patients over this time has generated considerable pharmacokinetic and pharmacodynamic data. This information should be useful for setting up clinical trials for this drug in neuroblastomas. Although actinomycin D is relatively well tolerated, hematological toxicities are observed in some of the children [42]. New drug combinations may provide a way to lower the effective chemotherapy doses in existing treatment protocols. In this study we show that, in addition to its tumor suppressive activity as a single agent, actinomycin D synergizes with the histone deacetylase inhibitor, SAHA in vitro on neuroblastoma cells.

SAHA was the first histone deacetylase inhibitorm approved by the US Food and Drug Administration, and phase II clinical trials in children with relapsed solid tumors including neuroblastoma are currently ongoing [26, 31, 43]. Histone deacetylase inhibitors have been shown to induce G1-phase cell cycle arrest, associated with the upregulation of p21 ^Waf1/Cip1^, independently of p53 [44, 45]. These changes result in reduced proliferation, induction of apoptosis and differentiation [46]. Although a number of clinical trials have been undertaken with SAHA, the efficacy of this drug as a single agent is low in neuroblastoma. The multiplicity of the genomic alterations found in neuroblastoma suggest that targeting multiple biological pathways are likely to be more effective than drugs that target a single pathway. Accordingly the use of SAHA, in combination with retinoic acid, results in improved anti-tumorigenic activity compared to either drug alone [47, 48]. The fact that p53 wild-type cells are more sensitive to actinomycin D, and that SAHA acts independently of p53, suggests this combination may be excellent for the treatment of neuroblastoma. Note that although most of the neuroblastomas present p53 wild-type, about 2 % of the cases have p53 mutations at diagnosis and around 15 % in the relapsed tumor [49]. According to this, the combinatory treatment of SAHA and actinomycin D could be very efficient in the treatment of p53-mutated relapsed tumors.

Inhibition of ribosome biogenesis, and specifically inhibition of RNA pol I, is a promising therapeutic option for cancer. Efforts are currently directed to develop new drugs in this direction. The small molecule CX-5461, an inhibitor of rDNA transcription, has been shown to selectively kill B-lymphoma cells in vivo while maintaining the viability of the wild type population [19]. Recently, several novel DNA intercalating agents have been identify by drug screen that repress RNA pol I activity by inducing the degradation of the RPA194 subunit of Pol I [50]. The results obtained here with actinomycin D suggest these novel therapeutic agents have potential against neuroblastoma.

## Conclusions

In summary, our data demonstrate that actinomycin D, at low concentrations, inhibits proliferation and induces cell death in neuroblastoma cell lines, as well as tumor regression in xenograft tumor models. We provide experimental evidence showing that apoptosis is the major cell death mechanism triggered by actinomycin D treatment in p53^wt^ cell lines and, to a less extent, in p53-deficient cell lines. Important, we show that higher N-Myc levels, an indicator of bad prognosis, sensitize neuroblastoma to actinomycin D. Our data highlights the therapeutic potential of actinomycin D and suggests that low doses of this drug could be used in combination with other agents to take advantage of its dependence on p53, but avoid its non-specific effects.

## Methods

### Cell culture and chemicals

SH-SY5Y cell line was purchased from American Type Culture Collection. LA1-55n, SK-N-JD and SK-N-AS were kindly supplied by Dr. Jaume Mora (Children’s Hospital Sant Joan de Déu, Barcelona) and SH-EP*Tet*/21 N by Dr. Manfred Schwab (Deutsches Krebsforschungszentrum, Heidelberg). Cell lines were cultured in RPMI 1640 media and supplemented with 10 % fetal bovine serum, 100 U/ml penicillin, 100 μg/ml streptomycin and 2 mM L-glutamine (GIBCO, Life Technologies). Conditional silencing of N-Myc expression in SH-EP*Tet*/21 N cells line was achieved by adding 1 μg/ml of tetracycline (Sigma-Aldrich) to growth media. Others chemicals used were actinomycin D (BioVision), nutlin-3 (Santa Cruz Biotechnology), SAHA (Cayman Chemical), and the pan-caspase inhibitor quinolyl-valyl-O-methylaspartyl-[−2,6-difluorophenoxy] [51] 7-methyl ketone (QVD-Oph, R&D Systems).

### Cell viability assays

3-(4,5-dimethylthiazol-2-yl)-5-(3-carboxymethoxyphenyl)-2-(4-sulfophenyl)-2H-tetrazolium (MTS) assay was carried out on cells seeded 24 hours prior to treatment on 96-well plates according to manufacturer’s instructions (Promega). Ratio of cell viability was calculated comparing the sample’s optical density to untreated controls at same time point. Cell number was calculated comparing the sample’s optical density to a standard curve. Assays were performed in triplicates.

### Quantitative real-time PCR

RNA was isolated using TRIzol (Life Technologies) according to manufacturer’s instructions. RNA was reverse transcribed using MMLV reverse transcriptase (Life Technologies) for 30 minutes at 37 °C. Real-time quantitative RT-PCR (qPCR) was performed using LightCycler 480 SYBR Green I Master kit (Roche) and the following primer sets (Sigma): MYCN forward 5′-TCCACCAGCAGCACAACTATG-3′ reverse 5′-GTCTAGCAAGTCCGAGCGTGT-3′; E2F1 forward 5′-ATGTTTTCCTGTGCCCTGAG-3′ and reverse 5′-ATCTGTGGTGAGGGATGAGG-3′; 45S rRNA forward 5′-CCCGTGGTGTGAAACCTTC-3′ and reverse 5′-GACGAGACAGCAAACGGGAC-3′; β-actin forward 5′-CGTCTTCCCCTCCATCG-3′ and reverse 5′-CTCGTTAATGTCACGCAC-3′. Calculation of relative mRNA was done using Light Cycler 96 software (Roche). Assays were performed in triplicates.

### Western blot

Protein extraction, separation and detection were achieved as described previously [52]. Antibodies used were: anti-PARP-1, anti-p53, anti-MDM2 and anti-p21 from Santa Cruz Biotechnologies; anti-N-Myc (Calbiochem), anti-LC3 (MBL International), anti-E2F1, anti-β − actin and γ-H2A.X (Cell Signaling Technology) and p73 (Abcam). The assays were repeated a minimum of three times.

### siRNAs and transfection

The following siRNAs were used: non-silencing siNT (GCAUCAGUGUCACGUAAUA) and p53 siRNA (GCATCTTATCCGAGTGGAA). Cells were transfected using lipofectamine 2000 according to the manufacturer instructions.

### Assessment of synergism

Synergism was calculated according to the Chou-Talalay median effect analysis and determined by the combination index (CI) [53]. Briefly, SK-N-JD and LA1-55n cell lines were treated with SAHA or actinomycin D individually at serial dilutions, or both simultaneously at fixed molar drug ratios of 1:500 (SK-N-JD) or 1:300 (LA1-55n) for 48 hours cells. The initial concentrations were 2.5 μM for SAHA and 1nM (SK-N-JD) or 20 nM (LA1-55n) for actinomycin D. Cell viability was then determined by MTS assay as previously described. The drug doses and their effect values were then used to determine whether the interaction was synergic (CI < 1), additive (CI = 1), or antagonic (CI > 1) using the CalcuSyn software v. 2.1 (Biosoft Inc.).

### Cell cycle analysis

Floating and adherent cells were collected after treatment, washed twice with cold PBS, fixed with ice cold 70 % ethanol, and centrifuged. Cells were then washed and resuspended in 1 ml of phosphate-buffered saline containing propidium iodide 5 μM and RNase A 100 μg/mL (Life Technologies). 10^4^ cells were then analyzed by flow cytometry (Beckman Coulter, Indianapolis, IN), and cell cycle distribution was determined by DNA content.

### In vivo treatment of Xenograft model

Six-week old female athymic mice (Harlan Laboratories) were used to propagate subcutaneously-implanted neuroblastoma tumors derived from SK-N-JD cells. Once tumours reached 15 mm at the largest axis, the donor mice were euthanized and equal-size pieces of the tumors were engrafted in both flanks of recipient mice. When tumors were palpable, the mice were divided into 4 cohorts of 8 mice each to receive, by intra-peritoneal injections, one of the following treatments: (a) vehicle control (PEG400 50 % in saline solution), (b) vorinostat (100 mg/kg per dose), (c) actinomycin D (60 μg/kg per dose), (d) vorinostat plus actinomycin D. Injections were given daily during a 1-week period, after which the dose for each treatment were halved and given for another week. Tumor measurements were obtained once every other day and converted to tumor volume using the equation Volume = (3,1416/6 × length × width^2^). Weights were measured daily. The mice were humanely killed at the end of the experiment. The in vivo experimental protocol was approved by the Committee of Animal Experimentation of the Catalonian Government.

### Histology and immunohistochemistry

Tumors were fixed in 10 % neutral formaldehyde, processed, and embedded in paraffin. Endogenous peroxidase activity was quenched by incubation of sections in 0.1 % hydrogen peroxide, and antigen retrieval was achieved using heat-activated 10 mM sodium citrate buffer (pH 6). Sections were incubated for 16 minutes at room temperature using antibodies against Ki67 (Ventana Medical Systems) and p53 (DAKO). All slides were counterstained with hematoxylin, dehydrated, and mounted.

### Statistical analysis

Results are expressed as means ± SEM of three separate experiments. Statistical significance of was determined by one-way Anova + Tukey test ( *p < 0.05, **p < 0.01 and ***p < 0.001).

## Additional files

Additional file 1: Figure S1. Cell cycle distribution after actinomycin D treatment. Indicated cell lines were treated with 10 nM of actinomycin D in the presence or in the absence of 20 μM of Oph-QVD for 24 h or 48 h. Cell cycle distribution was detected by the propidium iodide staining method and indicated in the histograms. (DOCX 866 kb) Additional file 2: Figure S2. Time course of H2A.X phosphorylation after SAHA and Actinomycin D treatment. LA1-55n cells were treated (+) or not (-) with 0.1 nM of actinomycin D (ActD) in the presence (+) or in the absence (-) of 1 μ M SAHA. Indicated protein expression was determined by Western blot analysis at the indicated times after the treatment. (DOCX 710 kb)
