# Supplementary material for: Effect of low doses of actinomycin D on neuroblastoma cell lines
Source: Mol Cancer. 2016 Jan 4;15:1. doi: 10.1186/s12943-015-0489-8 (PMC4698870; doi:10.1186/s12943-015-0489-8)
Supplement: Additional file 1: Figure S1. — Cell cycle distribution after actinomycin D treatment. Indicated cell lines were treated with 10 nM of actinomycin D in the presence or in the absence of 20 μM of Oph-QVD for 24 h or 48 h. Cell cycle distribution was detected by the propidium iodide staining method and indicated in the histograms. (DOCX 866 kb) [file 12943_2015_489_MOESM1_ESM.docx]

**
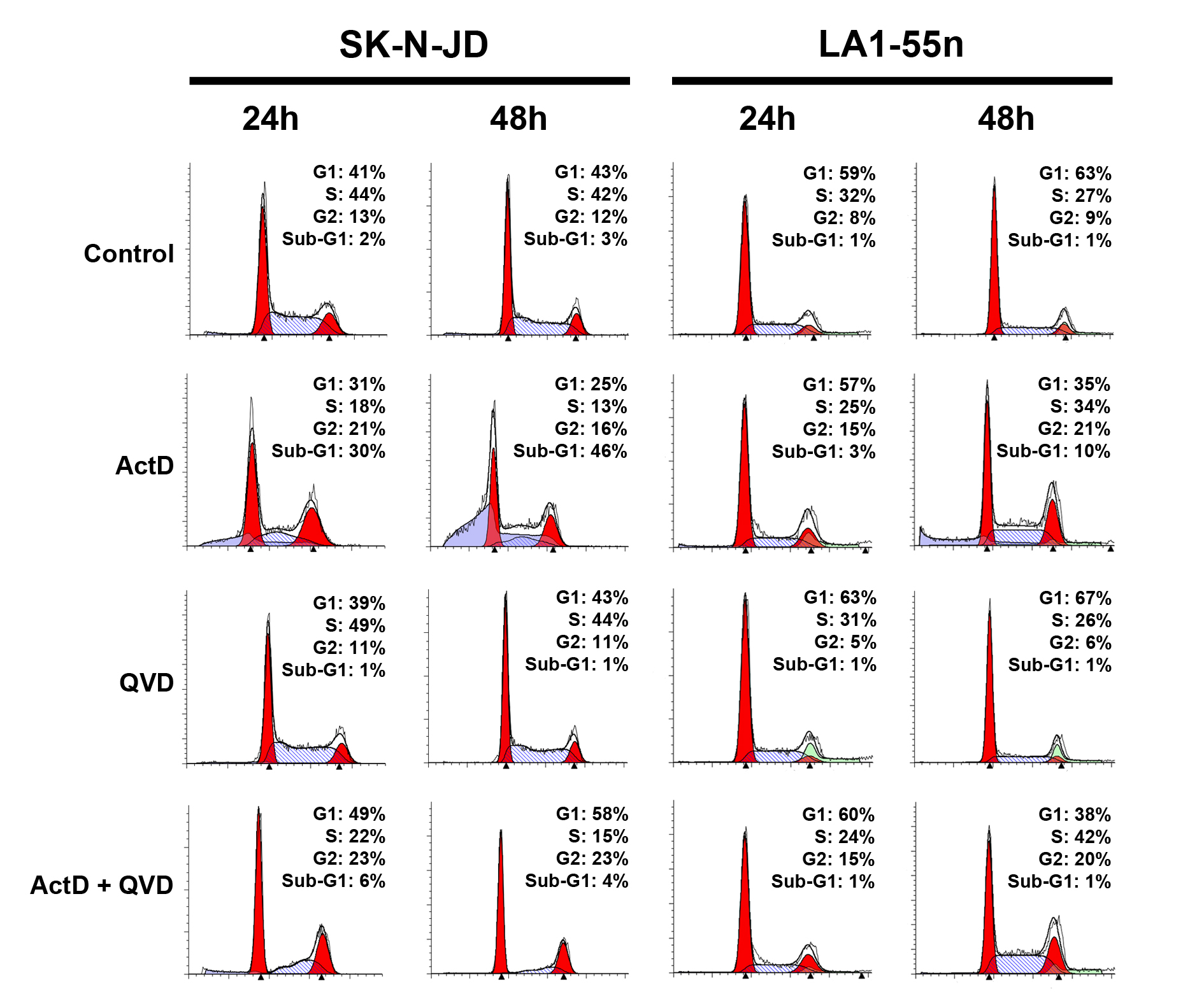
**

**Figure S1.- Cell cycle distribution after actinomycin D treatment.** Indicated cell lines were treated with 10 nM of actinomycin D in the presence or in the absence of 20 μM of Oph-QVD for 24 h or 48 h. Cell cycle distribution was detected by the propidium iodide staining method and indicated in the histograms.
