# Supplementary material for: Effect of low doses of actinomycin D on neuroblastoma cell lines
Source: Mol Cancer. 2016 Jan 4;15:1. doi: 10.1186/s12943-015-0489-8 (PMC4698870; doi:10.1186/s12943-015-0489-8)
Supplement: Additional file 2: Figure S2. — Time course of H2A.X phosphorylation after SAHA and Actinomycin D treatment. LA1-55n cells were treated (+) or not (-) with 0.1 nM of actinomycin D (ActD) in the presence (+) or in the absence (-) of 1 μ M SAHA. Indicated protein expression was determined by Western blot analysis at the indicated times after the treatment. (DOCX 710 kb) [file 12943_2015_489_MOESM2_ESM.docx]

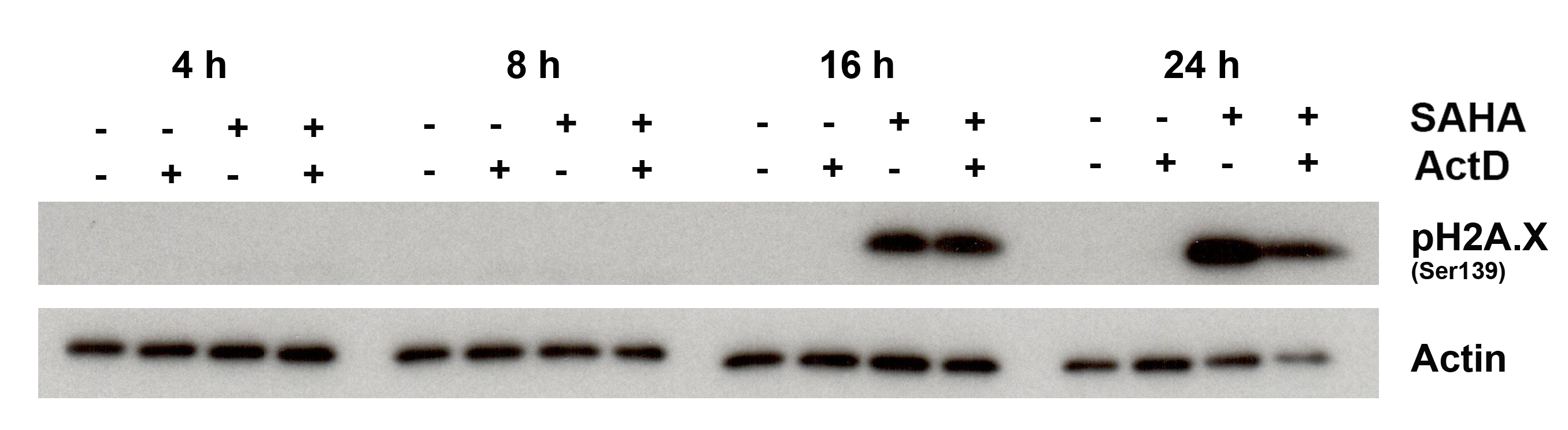


**Figure S2. Time course of H2A.X phosphorylation after SAHA and Actinomycin D treatment.** LA1-55n cells were treated (+) or not (-) with 0.1 nM of actinomycin D (ActD) in the presence (+) or in the absence (-) of 1 μ M SAHA. Indicated protein expression was determined by Western blot analysis at the indicated times after the treatment.
